# UTX/Top2β axis mediated spinal cord microvascular endothelial cells senescence exacerbates spinal cord injury

**DOI:** 10.1371/journal.pone.0338326

**Published:** 2025-12-12

**Authors:** Dongliang Liu, Yudong Liu, Tianding Wu, Jianzhong Hu

**Affiliations:** 1 Department of Emergency, Xiangya Hospital, Central South University, Changsha, Hunan, China; 2 First Department of Thoracic Surgery, Hunan Cancer Hospital and the Affiliated Cancer Hospital of Xiangya School of Medicine, Central South University, Changsha, Hunan, China; 3 Department of Spine Surgery and Orthopaedics, Xiangya Hospital, Central South University, Changsha, Hunan, China; Shanghai Jiao Tong University, CHINA

## Abstract

**Background:**

Cellular senescence plays a critical role in regulating angiogenesis. While UTX has been implicated in vascular regeneration following spinal cord injury (SCI), its underlying mechanisms remain incompletely understood. This study investigates how senescence mediates UTX-regulated vascular responses after SCI.

**Methods:**

We assessed p16^INK4a^ (a senescence marker) and UTX expression in spinal cord endothelial cells at 3, 5, 7 and 14 days post-SCI. By breeding UTX^flox/flox^ mice with heterozygous Tek-Cre mice, we generated endothelial-specific UTX knockout mice. Cellular senescence and proliferation were evaluated in primary spinal cord microvascular endothelial cells (SCMVECs). RNA sequencing and chromatin immunoprecipitation (ChIP)-seq were performed to identify UTX target genes.

**Results:**

UTX and p16^INK4a^ expression were both elevated post-SCI in a time-dependent manner. UTX deletion reduced endothelial senescence and enhanced proliferation both in vivo and in vitro. RNA-seq and ChIP-seq identified Top2β as a direct transcriptional target of UTX, negatively regulated through promoter binding. These findings were validated by ChIP-qPCR, western blotting, and immunofluorescence. Knockdown of Top2β reversed the anti-senescence and pro-proliferative effects induced by UTX deletion.

**Conclusions:**

UTX deletion attenuates vascular endothelial senescence and promotes angiogenesis after SCI by upregulating Top2β expression. This newly defined UTX/Top2β axis regulates vascular regeneration and represents a potential therapeutic target for promoting vascular repair following SCI.

## Introduction

Spinal cord injury (SCI) constitutes a devastating trauma to the central nervous system, resulting in the permanent loss of locomotor and sensory functions, as well as potential complications such as infection, cardiorespiratory failure, and even death [1,2]. The annual incidence of spinal cord injury (SCI) ranges from 1.2 to 5.8 cases per 100,000 population, with an estimated economic burden exceeding $9.7 billion annually [3]. The high incidence and morbidity rates of SCI present an unprecedented challenge to both society and the medical system.

Extrinsic and intrinsic factors compress or transect the spinal cord, disrupting the vasculature and blood–spinal cord barrier [4,5]. These events subsequently trigger sustained secondary injury cascades, including ischemia, inflammation, and oxidative stress, further damaging the spinal cord and neurological function [6,7]. Microvessel injury, considered one of the crucial pathological characteristics of SCI, occurs immediately after SCI. This disruption hampers the steady supply of nutrients and oxygen, exacerbating the progression of SCI pathogenesis [4,8]. Early intervention to repair the damaged microvascular system and promote angiogenesis post-SCI can effectively prevent secondary injury cascades and alleviate neurological deficits, thus improving the prognosis and overall life quality of patients [9,10].

While significant insights have been gained into the biological processes following SCI, progress in therapeutic options has been limited, indicating the potential importance of other cellular players in injury response. Cellular senescence, characterized by an irreversible halt in cell division [11], significantly impacts vascular function [12]. Senescent endothelial cells (ECs) exhibit impaired functions, including reduced nitric oxide secretion, which restrains cell proliferation [13]. Moreover, senescent ECs have diminished self-repair capacity, leading to their accumulation and the expression of senescence-associated secretory phenotypes. This accumulation promotes senescence in neighboring cells and contributes to progressive vascular dysfunction [14,15]. Notably, the emergence of senolytics, medicine designed to target and remove senescent cells, presents a promising avenue for reducing tissue damage and fostering regeneration in peripheral tissue wound healing process [16,17].

Cellular senescence is primarily regulated by two key pathways: the p16^INK4a^/Rb pathway and the p53/p21 pathway [18]. The p53–p21 axis is predominantly activated by DNA damage responses, whereas epigenetic alterations (e.g., promoter methylation, chromatin remodeling) often induce senescence via p16^INK4a^ upregulation [19,20]. Notably, as a critical component of histone demethylase complexes, UTX plays a central role in chromatin remodeling that influences gene expression and vascular function [21]. In our previous work, we demonstrated that EC-specific UTX deletion promotes vascular rejuvenation and enhances neural regeneration after SCI [22,23]. UTX has also been implicated in chondrocyte senescence, where its dysregulation accelerates cartilage degradation in osteoarthritis [24]. Given these findings, we hypothesize that UTX-mediated disruption of microvascular angiogenesis post-SCI may involve p16^INK4a^-related senescence mechanisms. In this study, we aim to investigate the role of cellular senescence and the mechanisms underlying UTX deletion-mediated vascular regeneration after SCI using endothelial-specific UTX knockdown mice.

## Materials and Methods

### Animal

C57BL/6J male mice were procured from the Laboratory Animal Centre of Xiangya Medical School (Hunan, China). UTX^flox/flox^ mouse (UTX^f/f^, stock, no. 021926) and Tek-Cre mouse (SJ-008863) were obtained from Jackson Laboratory and Shanghai Model Organisms, respectively. Heterozygous Tek-Cre mouse were intercrossed with UTX^f/f^ mouse to generate Tek-Cre; UTX^f/f^ mouse, denoting conditional UTX deletion in Tek lineage cells (UTX^-/-^). The animal study protocol was approved by Ethics Committee of Central South University for Scientific Research (approval No. LL2023030466). All procedures were in adherence with institutional guidelines and the Declaration of Helsinki.

### Contusion SCI model

We established the contusion SCI model using procedures described in our previous publication [22]. Briefly, mice were anesthetized by intraperitoneal injection of ketamine (80 mg/kg) and xylazine (10 mg/kg). Anesthesia depth was confirmed by absence of pedal reflex before proceeding. Under anesthesia, a T10 laminectomy was performed to expose the spinal cord. A moderate contusion injury was then induced using a modified Allen’s weight drop apparatus with a 10 g weight dropped from a height of 20 mm. Sham-operated mice underwent laminectomy without contusion. Postoperatively, buprenorphine and ketoprofen were administered for 48 hours to alleviate pain, and ampicillin was given for 3 days to prevent infection.

### Primary spinal cord microvascular endothelial cells (SCMVECs) culture

Primary SCMVECs were cultured following a previously published protocol with slight modifications [25]. Following euthanasia and decapitation, spinal column was quickly removed and placed in ice-cold isolation buffer (DMEM with 1% antibiotic-antimycotic). The spinal cord was then extracted by injecting cold isolation buffer through the sacral canal using a syringe. The dura mater was subsequently removed under a microscope. The spinal cord was washed with fresh isolation buffer and cut into ~1 mm pieces using a scalpel. Subsequently, the tissues were subjected to shaking in 0.1% collagenase II (Sigma, 9001-12-1) digestion buffer for 1.5 hours at 37°C. Digestion was halted by centrifugation (1000 rpm, 8 min), followed by resuspending pellets with 20% bovine serum albumin (BSA, Meilunbio, 9048-46-8) in DMEM. After centrifugation, pellets were further digested with 0.1% collagenase/dispase (Roche) under agitation for 30 minutes at 37°C (1000 rpm for 5 min). Pellets were then collected, washed, and resuspended in EC medium (LONZA, CC-4176) with 5% fetal bovine serum (Gibco) before being plated onto a rat tail collagen I-coated (Gibco, 10483−01) T25 flask for subsequent experiments.

### Flow cytometry analysis of primary SCMVECs

Isolated primary SCMVECs were harvested from the cell culture flask, and the total cell count was determined using an automated cell counter. CD31^+^ cells were isolated utilizing CD31 immunomagnetic beads (Miltenyi Biotec, 130-097-418) according to the instruction provided by the manufacturer. The selected CD31 microbead-positive cells were analyzed by flow cytometry with a CD31 antibody (1:100 in PBS containing 1% BSA). Cells were collected, washed, and centrifuged twice at 350g for 5 min at room temperature (RT). After CD31 antibodies incubation (4°C for 1 h), they were washed and centrifuged again. Afterward, the cells were resuspended in 50 μl PBS and subjected to flow cytometry (BD LSRFortessa™). Data were retrieved and analyzed using FlowJo software version 7.6.2.

### Immunofluorescence

Mouse were sacrificed after receiving a lethal dose of ketamine and xylazine anesthesia. Saline was perfused through the ascending aorta to remove blood, followed by harvesting and dehydrating a 5-mm spinal cord segment with the lesion epicenter for sectioning. After rewarming for 15 minutes at RT, frozen sections were then processed by washing with PBS, permeabilization with 1% Triton X-100, and blocking with 5% BSA in 1% PBS. For cell immunofluorescence experiment, primary SCMVECs underwent similar washing, fixation with 4% paraformaldehyde, permeabilization, and blocking steps, as described above. Primary antibodies (anti-CD31, 1:500 dilution, R&D, AF3628; anti-UTX, 1:200 dilution, Millipore, ABE409; anti-Top2β, 1:200 dilution, Proteintech, 20549–1-AP) were diluted in PBS containing 1% BSA and incubated overnight at 4 °C. After washing, appropriate fluorescent secondary antibodies diluted in the same buffer were applied for 1 hour at room temperature. Cells were then washed and mounted using Vectashield mounting medium with DAPI for confocal microscopy imaging (ZEISS LSM 900 with Airyscan 2, Germany).

### Cell viability assay

Primary SCMVECs at 3,000 cells/well in a 96-well plate were evaluated for cell viability using Cell Counting Kit-8 (CCK-8) per the manufacturer’s instructions (Dojindo, CK04–11). 10 μL of CCK-8 solution was added to the medium, incubated at 37°C for 2 hours, and absorbance at 450 nm measured with a microplate reader (TECAN, Switzerland).

### Western blot

SCMVECs were homogenized in RIPA buffer (Beyotime, China) with a proteinase inhibitor. Cell lysates were incubated on ice for 15 minutes and then centrifuged at 13,000 rpm for 15 minutes at 4°C. Protein concentrations in the supernatants were measured using a Bradford assay (Beyotime, P0006). After denatured at 100°C for 10 minutes, protein extracts (20−40 μg/well) were separated by 10% sodium dodecyl sulfate polyacrylamide gel electrophoresis, and transferred onto a polyvinylidene difluoride membrane (Millipore). Membranes were blocked with 5% nonfat dry milk in TBST (Tris-buffered saline with 0.1% Tween-20) for 1 hour at RT, then incubated overnight at 4 °C with primary antibodies diluted in the same blocking buffer: anti-UTX (1:1000 dilution, Millipore, ABE409), anti-Top2β (1:1000 dilution, ProteinTech, 20549–1-AP), p16^INK4a^ (1:1000 dilution, Abcam, ab211542), and β-tubulin (1:5000 dilution, ProteinTech, 66240–1-Ig). The following day, blots were visualized using the FluoChem M (ProteinSimple) after secondary antibody incubation (1:5000 dilution; ProteinTech) in 5% nonfat dry milk/TBST for 1 hour at RT, and subsequently quantified using ImageJ software.

### Senescence-associated β-galactosidase activity assay

SCMVECs from UTX^-/-^ and UTX^f/f^ were assessed for β-Galactosidase accumulation using a colorimetric senescence-associated β-galactosidase (SA-β-Gal) assay kit (Cell Signaling Technology, 9860S) per the manufacturer’s instructions. Briefly, 1 x 10^4^ SCMVECs were plated in a 24-well flat-bottomed plate with coverslips until confluence. After fixation at RT for 30 minutes with a 4% paraformaldehyde solution, the fixed cells were stained with fresh staining solution at pH at 6, 37°C overnight. The positively stained cells exhibiting a blue color were captured using a light microscope.

### Small interfering RNA (siRNA) transfection

Top2β siRNA (siTop2β) and NC siRNA (siNC) were provided by Ribo Biological Company, Guangzhou, China. SCMVECs were seeded in 6-well plates, and siRNA transfection was performed when cells reached approximately 50% confluence using the Ribo transfection kit (C10511-05). The oligonucleotide sequences of siRNAs were as follows: siNC:UUCUCCGAACGUGUCACGUTT;

siTop2β:GGUGUAUGAUGAAGAUGUAGG. Following manufacturer guidelines, siTop2β or siNC mixed with transfection reagents at 50 nM final concentration was applied to the cells for 48-hour incubation, followed by harvesting RNA and proteins for qRT-PCR and western blotting.

### RNA isolation and qRT-PCR

Spinal cord tissues from the injury site or cultured primary cells were collected. Total RNA was isolated using TRIzol Reagent (Invitrogen, 15596026CN) following the instructions of manufacturer. Extracted RNA was reverse-transcribed using the PrimeScript RT reagent Kit (Takara, RR037B) to generate complementary DNA (cDNA), followed by qRT-PCR analysis using the Arraystar SYBR® Green qRT-PCR Master Mix and a 7000 ABI detection system. Relative mRNA expression was assessed using the 2-ΔΔCt method with primer sets as follow: UTX-F: TGACTAAACTTCCTGCCTTCGTGAG; UTX-R: TTCTTGATGACCTGGTGTTCTGCTT, GAPDH-F:GATGCTGGTGCTGAGTATGTCG,GAPDH-R:GTGGTGCAGGATGCATTGCTCTGA.

### ChIp sequencing (ChIp-Seq)

The ChIP experiment was performed by KangChen Bio-tech (Shanghai, China). In brief, SCMVECs from UTX^f/f^ mice were crosslinked with 1% formaldehyde at RT for 10 min, quenched with glycine (0.25 mol/L), and lysed in SDS lysis buffer with a protease inhibitor cocktail. Genomic DNA was fragmented by sonication (30% power, 30s on and 30s off for 10 cycles; Diagenode, Denville, USA). Immunoprecipitation was done with UTX antibodies (1:50 dilution in ChIP dilution buffer; Cell Signaling Technology) overnight at 4°C, and chromatin-antibody complexes were pulled down with protein G magnetic beads (Thermo Fisher, 10004D). After reversing crosslinking, DNA was isolated for sequencing analysis using phenol-chloroform extraction. Library preparation and sequencing were performed using the TruSeq Nano DNA Sample Prep Kit (Illumina, FC-121–4002) and Illumina HiSeq 4000, respectively.

Raw reads were subjected to quality control using FastQC (v0.11.5) and adapter trimming using Trimmomatic (v0.36). Clean reads were aligned to the mm9 mouse reference genome using Bowtie2 (v2.1.0), and duplicate reads were removed with Picard (v1.92). Peaks were called using MACS (v1.4.2) with a p-value threshold of 1 × 10 ⁻ ⁴. Peak genomic distribution (promoter, exon, intron, intergenic, and upstream regions) was annotated using ChIPseeker (v1.18.0).

### ChIP-qPCR

The ChIP-qPCR experiment aimed to assess the binding of UTX to the Type II DNA Topoisomerases (Top2β) promoter. Both ChIP and input DNA were extracted. The DNA samples, along with Arraystar SYBR® Green qPCR Master Mix and primers for Top2β, were prepared for PCR reaction using the 7000 ABI detection system. The primers of Top2β were the following:

F:5’CCTGTCCCAGAGGAATGTGC3’,R:5’CTAGCCTGCTGTCAAAGTCGTC’. The Ct values of ChIP DNA fractions were normalized to Input DNA Ct values to determine the percentage of input.

### RNA sequencing (RNA-Seq)

The RNA-Seq sequencing experiment was conducted by KangChen Bio-tech (Shanghai, China). RNA was extracted from SCMVECs of UTX^-/-^ and UTX^f/f^ mice, with three biological replicates per group. High-quality RNA samples were used for library construction (150–300 ng) using the TruSeq RNA Sample Preparation Kit and sequenced on Illumina HiSeq 4000. Raw reads underwent quality control using FastQC and trimming using Trimmomatic. Clean reads were aligned to the mm9 reference genome using HISAT2 (v2.1.0), and gene expression was quantified using Ballgown (v2.10.0). Genes with FPKM < 0.5 were filtered out. Differential expression genes (DEGs) between UTX^⁻/⁻^ and UTX^^f^/^f^^ groups were analyzed using DESeq (Bioconductor) with thresholds of fold change > 1.5 or < 0.8 and false discovery rate < 0.05. Functional enrichment analysis, including Gene Ontology (GO) and KEGG pathways, was performed using clusterProfiler (v3.8.1). For GO and KEGG analyses, gene ratio (number of DEGs annotated to a term divided by total genes annotated to that term) and fold enrichment (observed versus expected DEGs counts per pathway) were calculated to assess the significance and magnitude of enrichment.

### Statistical analysis

All experiments were conducted with three or more duplicates, and values from multiple experiments are presented as mean ± standard error of mean (SEM). Statistical analysis included an unpaired two-tailed t-test for comparing two groups and One-way ANOVA with Tukey–Kramer post-hoc test for multi-group comparisons. GraphPad Prism version 9.0 (GraphPad Prism Software, La Jolla, Calif.) was used for statistical analyses, with significance set at p < 0.05.

## Results

### Elevated UTX and p16^INK4a^ expressions in SCMVECs of SCI mice

**Fig 1:**
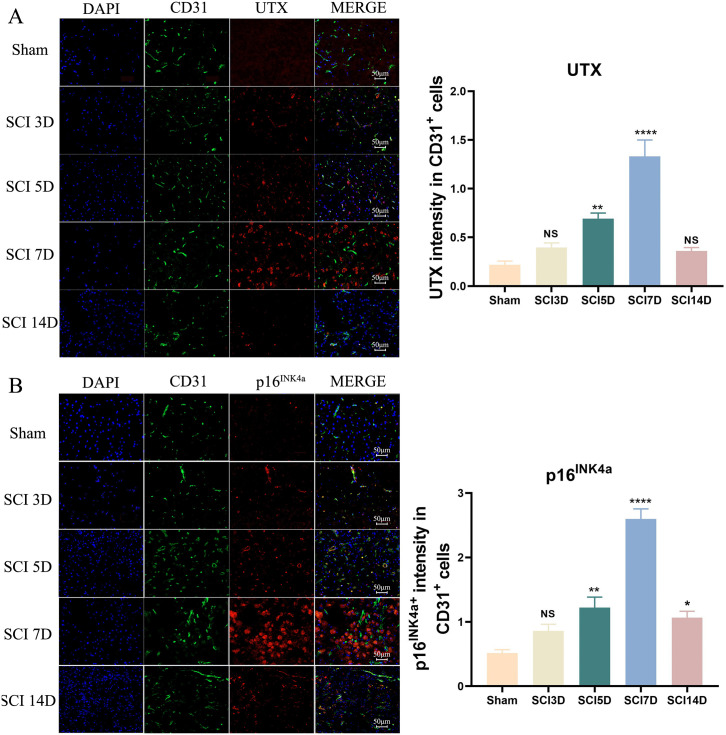
Elevated UTX and p16^INK4a^ expressions in SCMVECs of SCI mice. Representative immunofluorescence images and quantification of CD31^+^ (green), UTX^+^ (red) or p16^INK4a^+ (red), and DAPI (blue) in the injured spinal cord of mice at sham, 3, 5, 7, and 14 days post-SCI. Data are mean ± SEM; Scale bars: 50 μm. *p < 0.05, **p < 0.01, ****p < 0.0001 vs. sham. NS, not significant. SCMVECs, spinal cord microvascular endothelial cells; SCI, spinal cord injury.

To evaluate vascular alterations after SCI, we first quantified the CD31^+^ endothelial area relative to total nuclei (DAPI). We observed a reduction in CD31^+^ area at days 3, 5, and 7 post-injury, with a pronounced and statistically significant decrease by day 14 compared with sham controls (S1 Fig). This is consistent with the expected pathology, where robust infiltration of immune cells and gliosis leads to a relative decrease in the endothelial cell proportion, even amidst ongoing angiogenesis [26]. To investigate the potential involvement of vascular senescence in UTX-mediated responses to SCI, we examined the expression of UTX, p16^INK4a^ (canonical senescence marker), and CD31 (endothelial cell-specific marker) in the injured spinal cord of mice at days 3, 5, 7, and 14 post-injury. Immunofluorescence analysis demonstrated that UTX was predominantly localized in CD31^+^ endothelial cells, although a modest increase in UTX expression was also observed in CD31^−^ populations. Compared to sham controls, UTX expression was significantly elevated in a time-dependent manner after SCI (Fig 1A). A parallel temporal pattern was observed for p16^INK4a^, whose expression increased in concert with UTX (Fig 1B), suggesting a potential association between UTX upregulation and endothelial senescence during SCI.

To further explore this relationship, we assessed UTX and p16^INK4a^ expression in the spinal cords of aged (18-month-old) and young (2-month-old) mice using immunofluorescence staining. Both markers were significantly upregulated in SCMVECs of aged mice relative to young controls (S2 Fig), reinforcing a mechanistic link between UTX and endothelial aging. Taken together, these findings demonstrate that UTX and p16^INK4a^ are co-upregulated in SCMVECs under both pathological (SCI-induced) and physiological (age-related) conditions, implicating UTX as a potential regulator of vascular endothelial senescence and dysfunction in the injured spinal cord.

### Endothelial-specific UTX knockout decreased spinal cord vascular senescence in vivo

**Fig 2:**
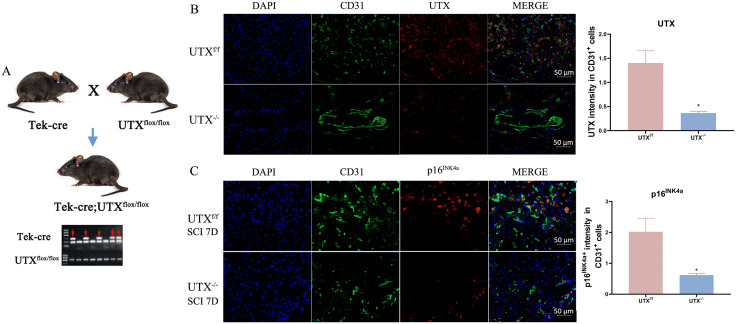
Endothelial UTX deletion attenuates vascular senescence after SCI. (A) Schematic of transgenic mice generation. (B) CD31^+^ (green), UTX^+^(red), and DAPI (blue) staining in spinal cords of UTX^-/-^ and UTX^f/f^ mice. (C) CD31^+^ (green), p16^INK4a^ (red), and DAPI (blue) staining in the spinal cord injury region of UTX^-/-^ and UTX^f/f^ mice at 7 day post SCI. Data are mean ± SEM; Scale bars: 50 μm. *p < 0.05 vs. UTX^f/f^. SCI, spinal cord injury.

We next generated transgenic UTX^-/-^ mice with specific UTX knockout in SCMVECs and their UTX^f/f^ littermate controls (Fig 2A). Efficient deletion of UTX in SCMVECs was confirmed by immunofluorescence staining (Fig 2B). Quantitative analysis of CD31^+^/DAPI staining in spinal cord sections revealed a borderline significant increase in vascular density in UTX^−/−^ mice compared to UTX^f/f^ controls (p = 0.05; S3 Fig). Subsequently, the expression of p16^INK4a^ and CD31 were examined at 7-day post-SCI utilizing a contusion SCI model. Our results indicate that UTX deletion significantly reduced p16^INK4a^ expression in SCMVECs relative to control mice (Fig 2C). These results underscore the vital role of UTX in driving vascular senescence after SCI.

### Endothelial-specific UTX knockout decreased cellular senescence and promoted cell proliferation in vitro

**Fig 3:**
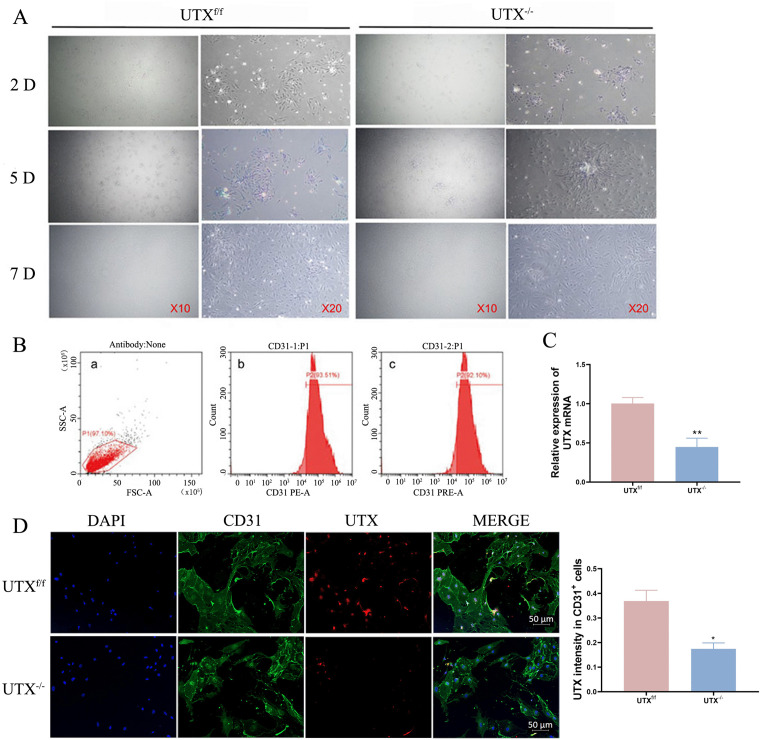
Primary SCMVECs Culture and Validation. (A) Phase-contrast images of primary SCMVECs from UTX^f/f^ and UTX^-/-^ mice. (B) Flow cytometry showing >90% CD31^+^ purity in UTX^f/f^ mice. (C) qRT-PCR analysis of UTX mRNA from UTX^f/f^ and UTX^-/-^ mice. (D) Representative images and quantitative analyses of CD31^+^ (green), UTX^+^(red), and DAPI (blue) in cultured primary SCMVECs from UTX^f/f^ and UTX^-/-^ mice. Data are mean ± SEM. Scale bars: 50 μm. *p < 0.05, **p < 0.01 vs. UTX^f/f^.

Isolating pure populations of primary SCMVECs from the spinal cord, especially from transgenic mice, is a crucial resource to explore the intricate molecular mechanism processes of vascular regeneration post SCI [27]. In this investigation, primary SCMVECs were isolated following a published protocol with slight modifications [25]. As shown in Fig 3A, images under phase-contrast microscopy displayed similar cell morphology between SCMVECs from UTX^-/-^ and UTX^f/f^ mice (Fig 3A). Flow cytometry confirmed that over 90% of isolated SCMVECs were CD31⁺ (Fig 3B). qRT-PCR of the same population showed an approximately 50% reduction of UTX mRNA in UTX^-/-^ SCMVECs compared with UTX^f/f^ controls (Fig 3C), reflecting partial recombination of the floxed allele in endothelial cells. UTX and CD31 staining further demonstrated the specific knockout of UTX in SCMVECs from the spinal cord (Fig 3D). All these results ensured the reliability of further exploring the mechanism of vascular regeneration disruption post SCI.

**Fig 4:**
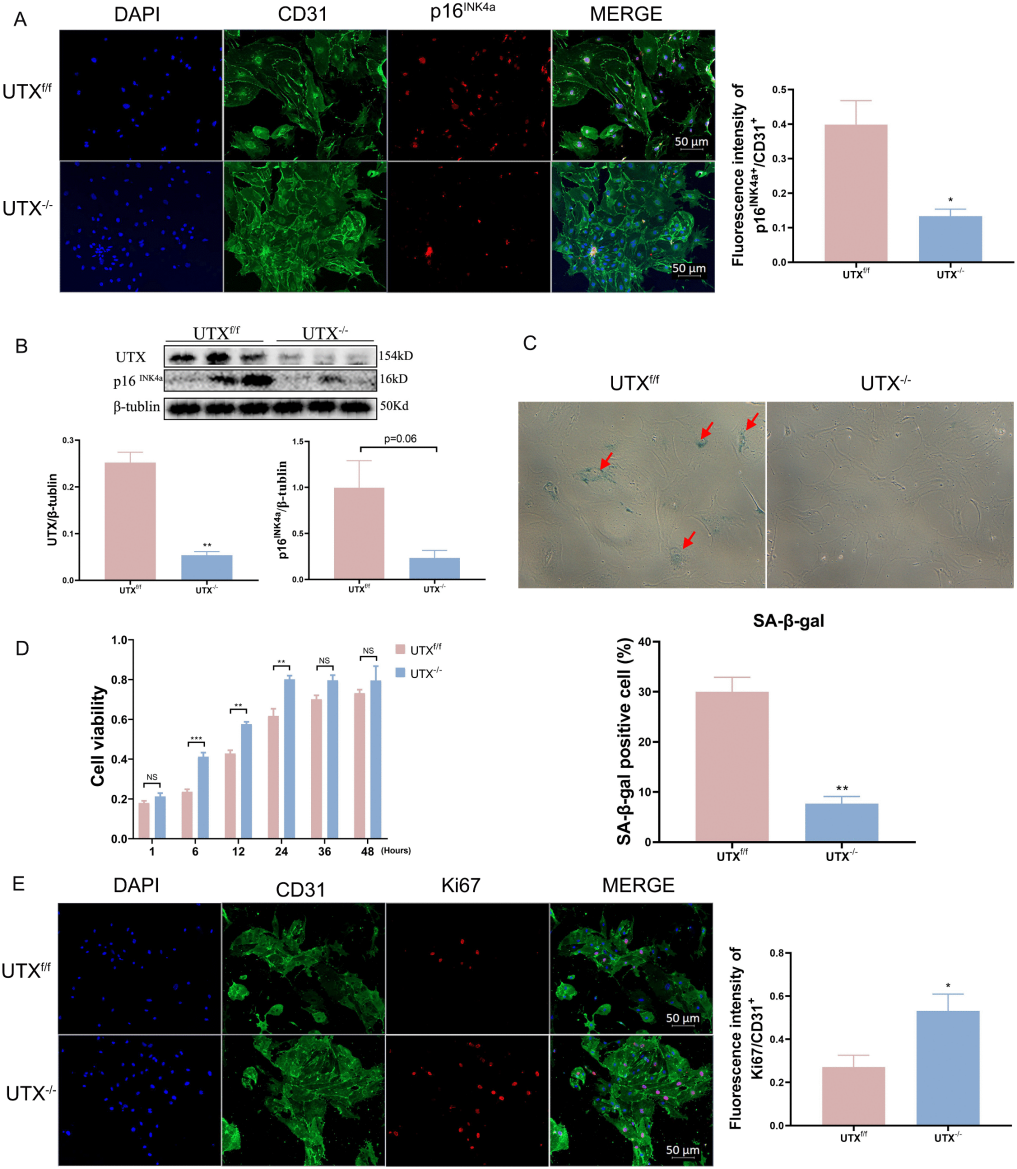
Endothelial UTX deletion reduces senescence and enhances proliferation in primary SCMVECs after SCI. Representative images and quantification of CD31^+^, p16^INK4a^ (red), and DAPI (blue) (A); immunoblotting of p16^INK4a^ and UTX (B); SA-β-gal staining (C); CCK-8 cell viability assay (D); and CD31^+^, Ki67^+^ (red), and DAPI (blue) staining (E) in primary SCMVECs from UTX^f/f^ and UTX^-/-^ mice. Data are mean ± SEM; scale bars: 50 μm. *p < 0.05, **p < 0.01 vs. UTX^f/f^ group. SCMVECs, spinal cord microvascular endothelial cells; SCI, spinal cord injury.

We next isolated SCMVECs from UTX^−/−^ and UTX^f/f^ mice at 7 days post-SCI to examine the levels of cellular senescence and proliferation in primary cultured SCMVECs. Our results showed that UTX deficiency significantly reduced cellular senescence, as evidenced by decreased p16^INK4a^ immunofluorescence, reduced protein levels, and diminished SA-β-gal staining (Fig 4A–C). Concurrently, Ki67 immunostaining and CCK-8 assays demonstrated enhanced proliferative activity in SCMVECs from UTX^−/−^ mice compared to UTX^f/f^ controls (Fig 4D–E). These findings underscore the critical role of UTX in regulating endothelial senescence and proliferation in the context of SCI.

### Identification of DEGs of UTX by RNA-seq

**Fig 5:**
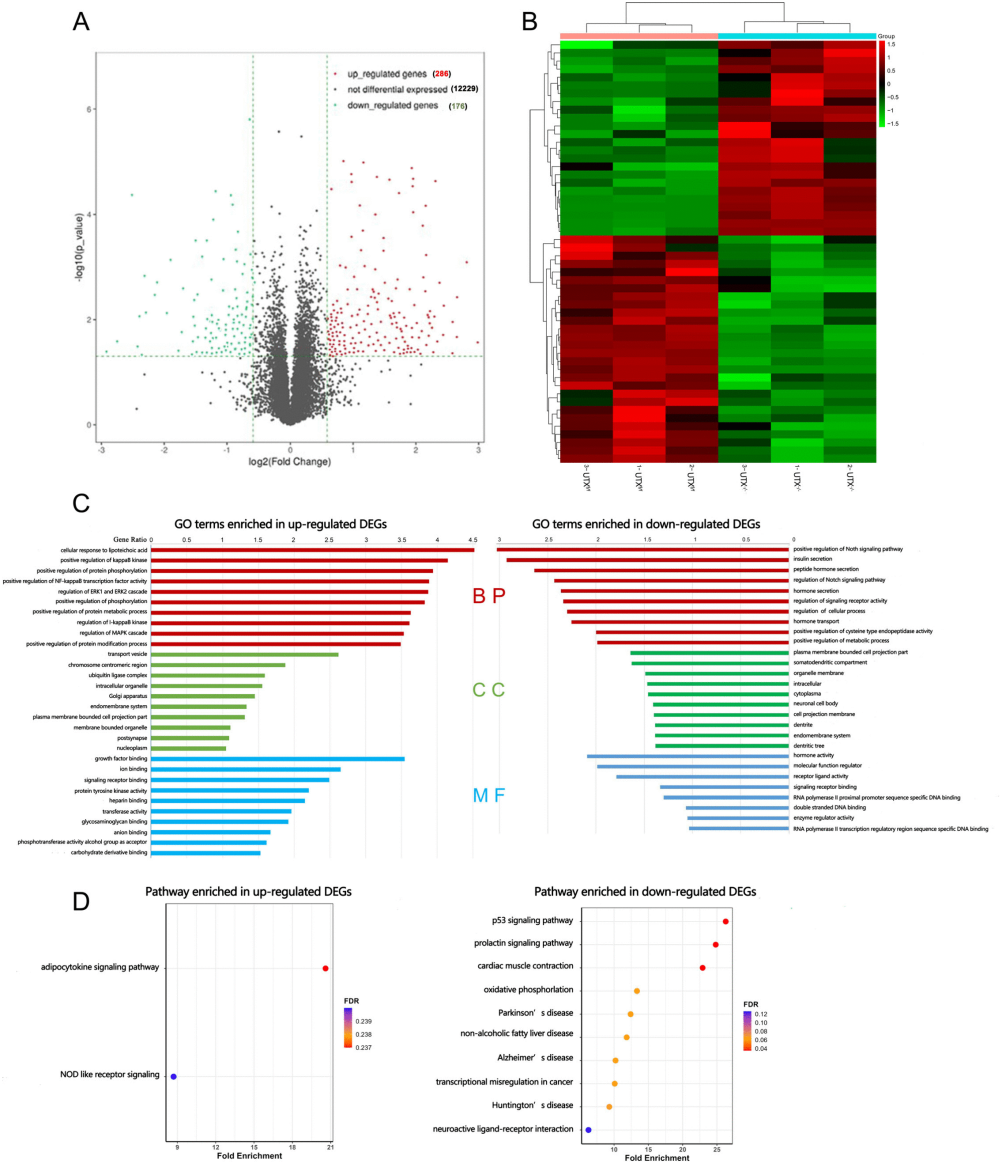
Transcriptomic profiling of UTX-deficient SCMVECs. (A) Volcano plot of differentially expressed genes (DEGs) between UTX^f/f^ and UTX^-/-^ groups. Red: upregulated; green: downregulated; black: not significant. (B) Heat map of DEGs. (C) GO analysis of up- and down-regulated DEGs. The x-axis shows the gene ratio (DEGs in a GO term/total genes in that term). (D) KEGG pathway analysis of up- and down-regulated DEGs. The x-axis shows fold enrichment, and bubble color represents false discovery rate.

To elucidate the potential molecular signaling targets of UTX, total RNA was extracted, and RNA-seq analysis was performed based on our primary cultured SCMVECs from UTX^-/-^ and UTX^f/f^ mice. RNA-seq transcriptome analysis identified 462 differentially expressed mRNAs, with 286 mRNAs upregulated and 176 mRNAs downregulated in SCMVECs of UTX^-/-^ compared to SCMVECs from UTX^f/f^, as shown in the volcano plot (Fig 5A). Cluster analysis revealed a significantly differential gene expression profile between the two groups (Fig 5B). The DEGs were subjected to GO analysis across three categories: molecular function, biological process, and cellular component. Significant enrichment was observed for molecular function terms such as growth factor binding, ion binding, and molecular function regulator; biological process terms including regulation of I-kappaB kinase/NF-kappaB signaling, NF-kappaB transcription factor activity, and Notch signaling pathway; and cellular component terms such as transport vesicles, chromosome centromeric regions, and plasma membrane-bounded cell projection parts (Fig 5C). KEGG pathway analysis revealed that DEGs were significantly enriched in pathways including senescence-related p53 signaling, prolactin signaling, and oxidative phosphorylation, among others (Fig 5D).

### Combination of RAN-seq and ChIP-seq identifying Top2β as UTX target gene related to cellular senescence

**Fig 6:**
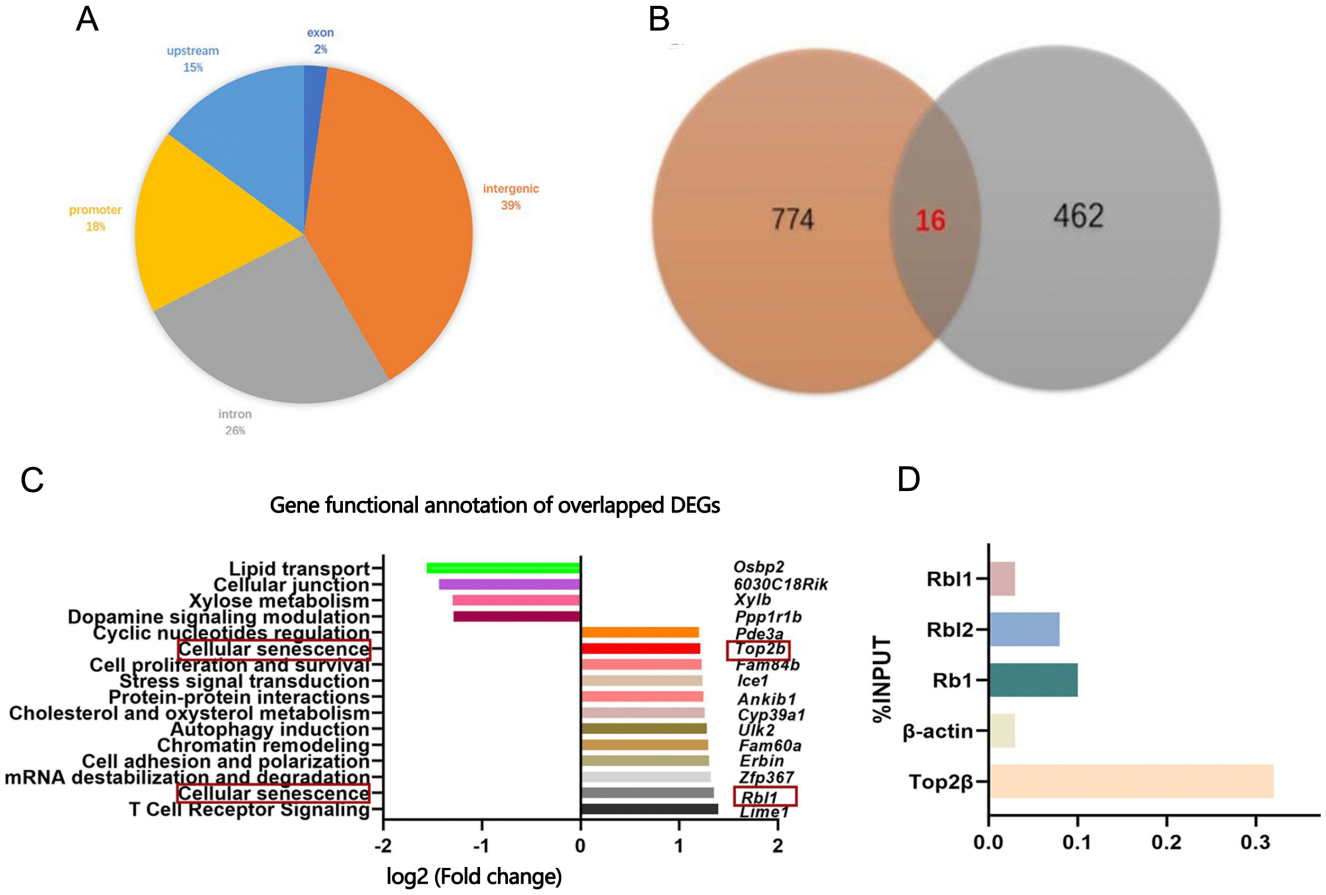
Integrated RNA-seq and ChIP-seq identify Top2β as a UTX target linked to senescence. (A) Pie chart showing genomic distribution of UTX binding sites from ChIP-seq. (B) Venn diagram showing overlap between differentially expressed genes (DEGs) from RNA-seq and genes with direct UTX promoter binding. (C) Functional annotation of overlapping DEGs; downregulated genes are shown on the left, upregulated genes on the right, with senescence-related genes highlighted in red. (D) ChIP-qPCR (% input) for UTX binding to Top2β, positive controls (RbI2, Rb1), and negative control (β-actin).

Through ChIP-seq, we next identified 4390 direct UTX binding sites, distributed across exons (2%, 99), intergenic regions (39%, 1724), introns (26%, 1140), promoter regions (18%, 774), and upstream areas (15%, 653) (Fig 6A). Given the pivotal role of promoter regions in regulating gene transcription in response to stimuli, we intersected the 462 DEGs identified by RNA-seq with the 774 genes whose promoter regions were bound by UTX, as determined from ChIP-seq data. This analysis revealed 16 DEGs with UTX-bound promoters supported by both assays (Fig 6B). Among these, the down-regulated genes included Osbp2, 6030419C18Rik, Xylb, and Ppp1r1b, while the up-regulated genes comprised Pde3a, Top2β, Fam84b, Ice1, Ankib1, Cyp39a1, Ulk2, Fam60a, Erbin, Zfp367, Rbl1, and Lime1, all showing altered expression due to UTX deletion. Further gene functional annotation revealed that only Top2β and Rbl1 were genes related to cell senescence (Fig 6C).

Based on previous reports, Minoru et al. identified Rb and Rbl2, but not Rbl1, as UTX targets directly activated by UTX expression [28]. Therefore, we focused our analysis on Top2β. ChIP-qPCR results showed that the binding affinity of UTX to the Top2β promoter was 0.32%, which was higher than that for Rbl2 (0.09%), Rb (0.11%), Rbl1 (0.031%), and the negative control β-actin (0.029) [28] (Fig 6D). These findings confirm a direct interaction between UTX and the Top2β promoter region.

### UTX deletion reduced senescence and promoted vascular regeneration by upregulating Top2β

**Fig 7:**
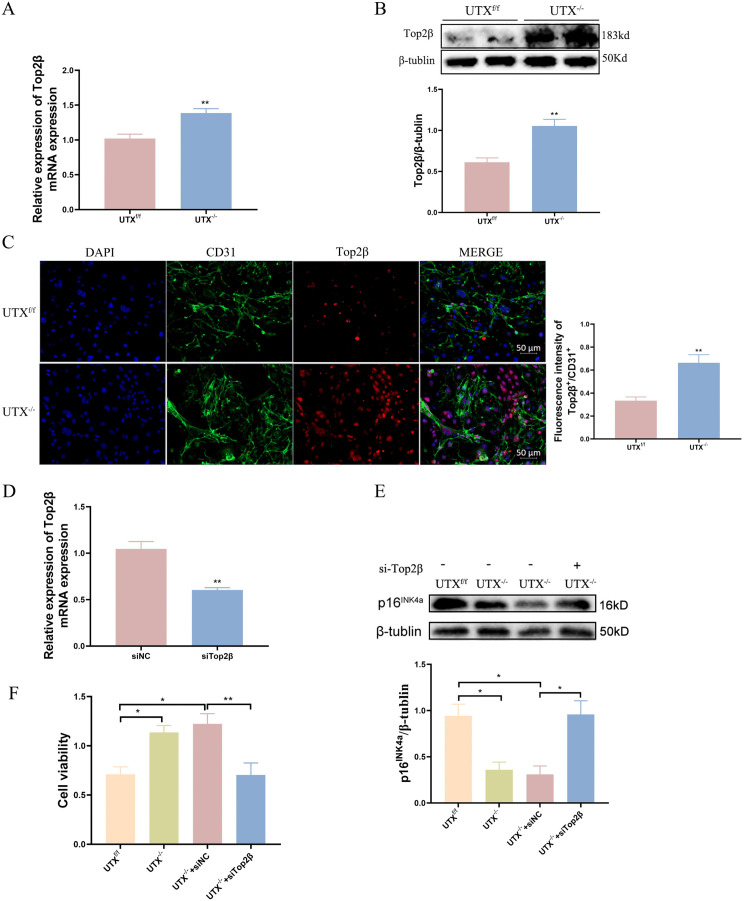
UTX deletion reduced senescence and promoted vascular regeneration by upregulating Top2β. (A) qRT-PCR analysis of Top2β mRNA; (B) Immunoblotting of Top2β protein; (C) Immunofluorescence of CD31^+^ (green), Top2β^+^ (red), and DAPI (blue) nuclei in UTX^f/f^ and UTX^-/-^ SCMVECs. (D)Relative Top2β mRNA expression in siNC vs. siTop2β groups by qRT-PCR. (E) Representative immunoblots and quantitative analysis of p16^INK4a^ protein in indicated groups. (F) Cell viability of SCMVECs in indicated groups. Data represent means ± SEM; *p < 0.05, **p < 0.01 vs. UTX^f/f^ group.

We then determined Top2β expression in our cultured primary SCMVECs from UTX^f/f^ and UTX^-/-^ mice. As shown in Fig 7A, qRT-PCR experiment demonstrated that UTX deletion in SCMVECs significantly unregulated Top2β mRNA expression (Fig 7A), which was corroborated by western blotting and immunofluorescence staining experiments (Figs 7B and 7C). Furthermore, we transfected SCMVECs with siRNA to knockdown Top2β expression (Fig 7D). Our findings indicated that Top2β knockdown could counteract the effects of UTX deletion, specifically the downregulation of p16^INK4a^ and promotion of cell viability, as depicted in Figs 7E-7F. These findings indicate that Top2β deletion reversed UTX deletion–induced anti-senescence and pro-proliferative effects in primary SCMVECs. The schematic summarizes the proposed mechanism by which the UTX/Top2β axis regulates SCMVEC senescence after SCI (S1 File).

## Discussion

In this study, we observed increased UTX and p16^INK4a^ expression in SCMECs following SCI. Further investigations demonstrated that mice with endothelial-specific UTX deletion showed reduced p16^INK4a^ levels in the injured spinal cord post-SCI in comparison to mice of UTX^f/f^. In vitro, UTX deletion in SCMECs decreased p16^INK4a^ expression and enhanced cell proliferation. Subsequent RNA-seq and ChIP-seq analyses identified Top2β as a potential target through which UTX mediates cellular senescence by binding to its promoter regions. Knockdown of Top2β could rescue UTX deletion induced senescence inhibition and proliferation promotion. These findings suggest that the protective effects of endothelial UTX deletion on vascular proliferation may be mediated, at least in part, by its regulation of vascular senescence via Top2β signalling.

Vascular EC damage occurs instantly after SCI, triggering early neovascularization [29]. However, the inherent vascular regeneration capacity falls short, leading to a marked decrease in vessel regeneration over time [30,31]. This insufficiency causes sustained ischemia and hypoxia in the injured area, resulting in neuronal cell death and irreversible neural function loss [6]. The progression of senescence, particularly its stages, might plays a crucial role in this context. Research shows that in high-regeneration models like zebrafish hearts, a temporary surge of senescent cells post-injury is beneficial for regeneration [32,33]. However, persistent senescence contributes to chronic diseases and aging-related disorders like atherosclerosis and neurodegeneration [34,35]. Consistent with these observations, recent studies underscore the complex role of senescence in cancer, where it may act as a barrier to tumorigenesis yet also contribute to disease progression and therapeutic resistance, depending on the biological context [36]. In our study, senescence peaked at 7 days post-SCI and gradually declined thereafter, suggesting that temporally selective inhibition of senescence during the subacute phase may offer therapeutic benefit. However, the long-term consequences of senescence on spinal cord repair and functional recovery remain to be elucidated.

Endothelial senescence plays a key role in vascular impairment [15,37]. Senescent ECs exhibit diminished proliferative capacity and reduced angiogenic potential, thereby limiting tissue repair. Moreover, senescent ECs adopt a senescence-associated secretory phenotype, characterized by the release of pro-inflammatory cytokines and chemokines that disrupt angiogenesis, compromise vascular integrity, and increase endothelial permeability and stiffness [38,39]. Notably, Chen et al. demonstrated that senolytic treatment attenuates EC senescence and enhances angiogenesis within the marrow space of the bony endplate [39]. In the present study, we also show that UTX deletion reduces endothelial senescence and promotes vascular regeneration, primarily through the upregulation of Top2β. These findings provide novel mechanistic insights into SCI pathogenesis and suggest that targeting UTX/Top2β mediated senescence represents a promising therapeutic strategy for promoting vascular repair after SCI.

UTX has emerged as a key regulator of cellular senescence, partly through its histone demethylase activity that removes the repressive H3K27me3 mark to promote gene transcription [40]. In C. elegans, increased UTX expression during aging is associated with global reductions in H3K27me3, whereas UTX knockdown restores H3K27me3 levels and extends lifespan, suggesting a functional link between UTX activity and senescence [41]. In osteoarthritis, UTX depletion alleviates chondrocyte senescence and alters mitochondrial and developmental gene programs [24]. Similarly, UTX overexpression promotes SA-β-gal activity and has been linked to the regulation of cancer stem cell behavior [42]. Mechanistically, senescent cells exhibit extensive chromatin remodeling, including the formation of senescence-associated heterochromatin foci [43], along with large-scale redistribution of H3K27me3 into enriched “mesas” and depleted “canyons” [44]. Collectively, these findings underscore UTX as a pivotal epigenetic regulator in cellular senescence.

To further elucidate the mechanisms by which UTX regulates endothelial senescence, we performed RNA-seq and ChIP-seq analyses on primary SCMVECs. This integrative approach identified Top2β as a direct transcriptional target of UTX, with ChIP-seq data confirming UTX binding at the Top2β promoter, thereby repressing its expression. Top2β, an ATP-dependent topoisomerase, is known to modulate chromatin architecture and epigenetically regulate genes involved in neuronal differentiation and longevity [45,46]. Consistent with our findings, Bhanu et al. reported reduced Top2β expression in senescent cerebellar granule neurons, implicating its relevance in aging-related processes [47]. In our study, UTX deletion led to Top2β upregulation and a concomitant reduction in endothelial senescence. Moreover, knockdown of Top2β reversed the anti-senescent effects of UTX deficiency, further substantiating Top2β as a critical mediator of UTX-driven vascular senescence. However, it should be noted that UTX operates within a complex epigenetic landscape involving various chromatin modifiers and transcription factors [48,49]. Xu et al. reported that UTX exerts limited influence on H3K27me3 but significantly enhances H3K27ac and chromatin accessibility in neural stem cells, thereby upregulating gene expression such as AP-1 [50]. Although UTX is classically recognized for promoting gene expression via H3K27me3 demethylation, our observation that Top2β is upregulated upon UTX deletion suggests that H3K27ac may play a more prominent regulatory role in this context. Further investigation is warranted to delineate the precise epigenetic mechanisms through which UTX modulates Top2β expression.

The following experimental limitations in this study need to be considered. First, we only used a modified Allen’s weight drop apparatus to establish the SCI model; Multiple SCI models, such as ischemia-reperfusion and spinal cord transection injury, are needed to further consolidate our observations. Second, although we have identified Top2β as the target of UTX, continued and upcoming research is necessary to explore the specific mechanism by which UTX regulates Top2β, affecting vascular angiogenesis post-SCI. Third, our in vitro results demonstrate that TOP2β knockdown reverses the p16^INK4a^ downregulation induced by UTX deletion, which suggests that TOP2β partially mediates UTX-related senescence effects; however, confirmation of these findings in vivo remains lacking and represents an important direction for future studies. Fourth, while our study elucidates the role of the UTX/Top2β axis in vascular senescence post-SCI, it does not address potential systemic or extrinsic factors—such as overall organismal aging or circadian rhythm dysregulation—that may also impact vascular regeneration and functional recovery [51,52], warranting further investigation.

In conclusion, this study demonstrated that UTX deletion inhibits cellular senescence of SCMVECs and promotes vascular proliferation post-SCI through upregulation Top2β. Our findings indicate the therapeutic potential of targeting vascular senescence to promote angiogenesis by mediating the UTX/Top2β axis in the context of SCI, providing further evidence for targeted pharmacological intervention.

## Supporting information

S1 Fig Reduced CD31^+^ endothelial area in the spinal cord after SCI. Representative images and quantitative analysis of CD31^+^ (green) and DAPI (blue) staining in the injury region of C57BL/6J mice at sham, and 3, 5, 7, and 14 days post-SCI. Data are presented as mean ± SEM; Scale bars: 50 μm. *p < 0.05 vs. sham group. SCI, spinal cord injury.
(TIF)

S2 Fig Elevated UTX and p16^INK4a^ expressions in SCMVECs of aged mice. Representative images and quantitative analyses depict CD31^+^ (green), UTX+(red)/p16^INK4a+^(red) and DAPI (blue) in the SCMVECs of aged (18-months) compared to young mice (2-months). Data are mean ± SEM; Scale bars: 50 μm. *p < 0.05, **p < 0.01 vs. young group.
(TIF)

S3 Fig CD31^+^ endothelial area in the spinal cord of endothelial-specific UTX knockout mice. Representative images and quantitative analysis of CD31^+^ (green) and DAPI (blue) staining in the spinal cord of UTX^f/f^ and UTX^-/-^ mice. Data are presented as mean ± SEM; Scale bars: 50 μm.
(TIF)

S4 Fig Original western blot strips. (PDF)

S1 Table Source data for all findings. (XLSX)

S1 File Graphical Abstract. Schematic illustrating the proposed role of the UTX/Top2β axis in regulating spinal cord microvascular endothelial cell senescence following spinal cord injury.
(TIF)
