# Supplementary figures and images for: UTX/Top2β axis mediated spinal cord microvascular endothelial cells senescence exacerbates spinal cord injury
Source: PLoS One. 2025 Dec 12;20(12):e0338326. doi: 10.1371/journal.pone.0338326 (PMC12700428; doi:10.1371/journal.pone.0338326)

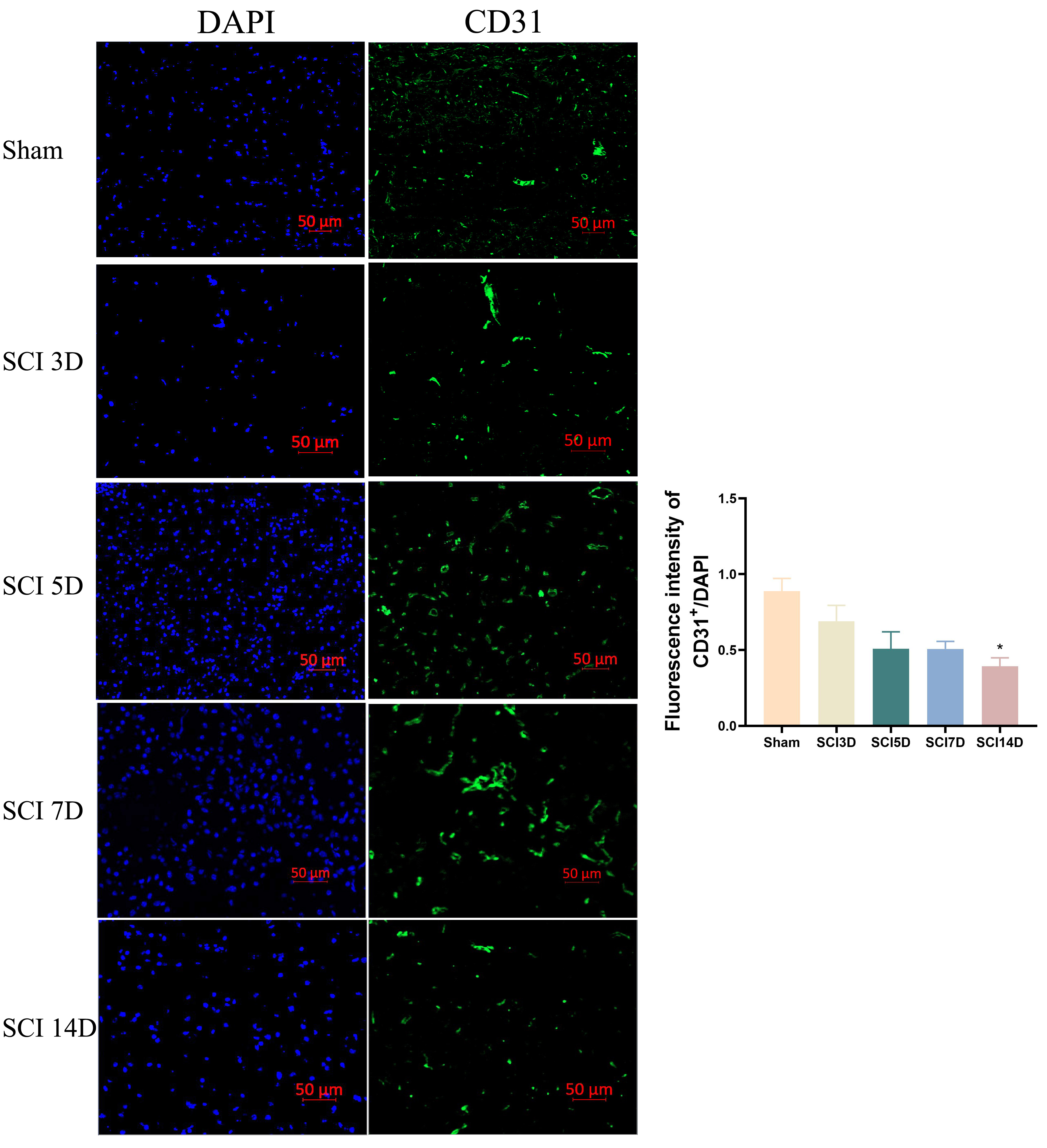

Supplement: S1 Fig — Representative images and quantitative analysis of CD31+ (green) and DAPI (blue) staining in the injury region of C57BL/6J mice at sham, and 3, 5, 7, and 14 days post-SCI. Data are presented as mean ± SEM; Scale bars: 50 μm. *p < 0.05 vs. sham group. SCI, spinal cord injury. (TIF) [file pone.0338326.s001.tif]

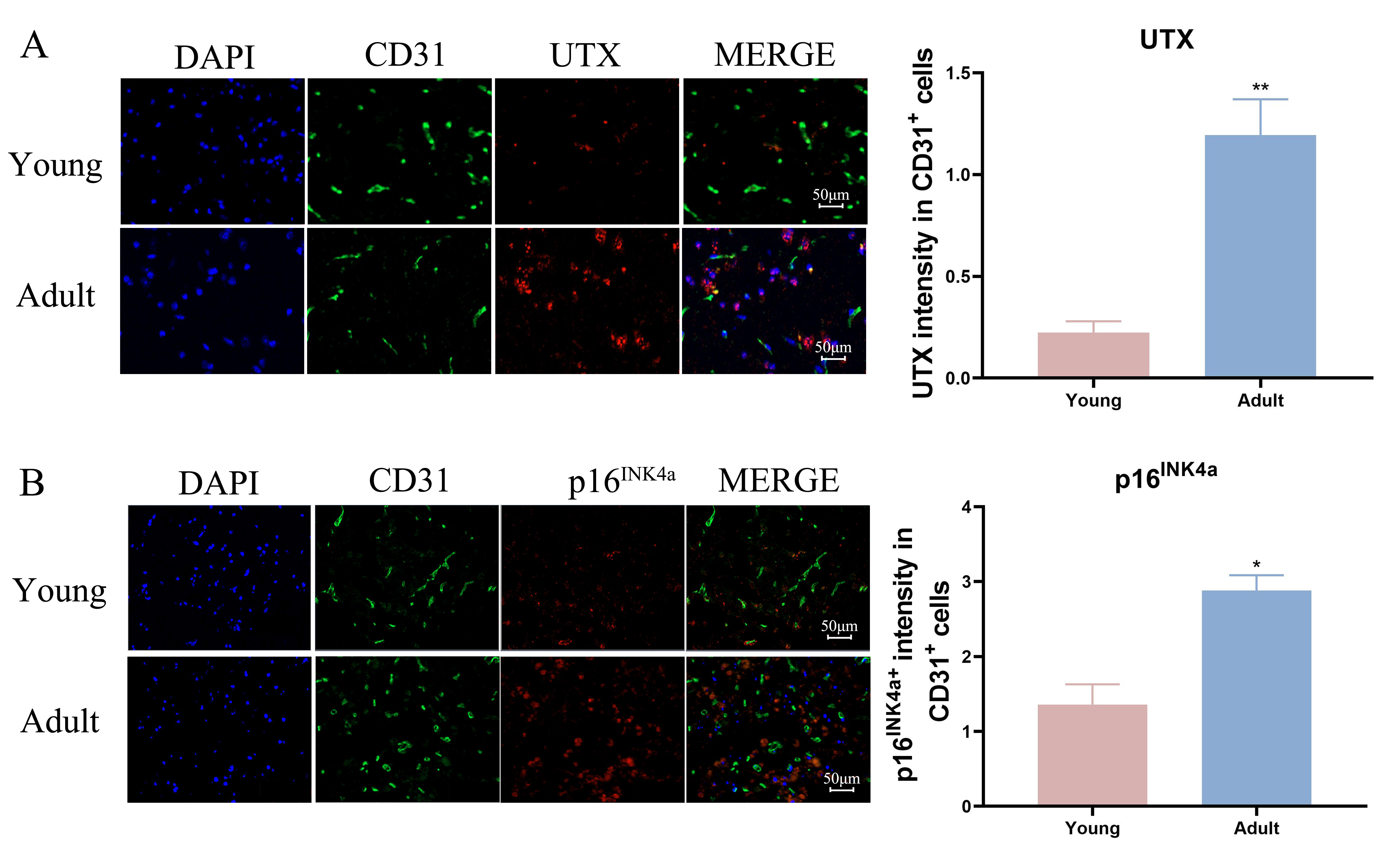

Supplement: S2 Fig — Representative images and quantitative analyses depict CD31+ (green), UTX+(red)/p16INK4a+(red) and DAPI (blue) in the SCMVECs of aged (18-months) compared to young mice (2-months). Data are mean ± SEM; Scale bars: 50 μm. *p < 0.05, **p < 0.01 vs. young group. (TIF) [file pone.0338326.s002.tif]

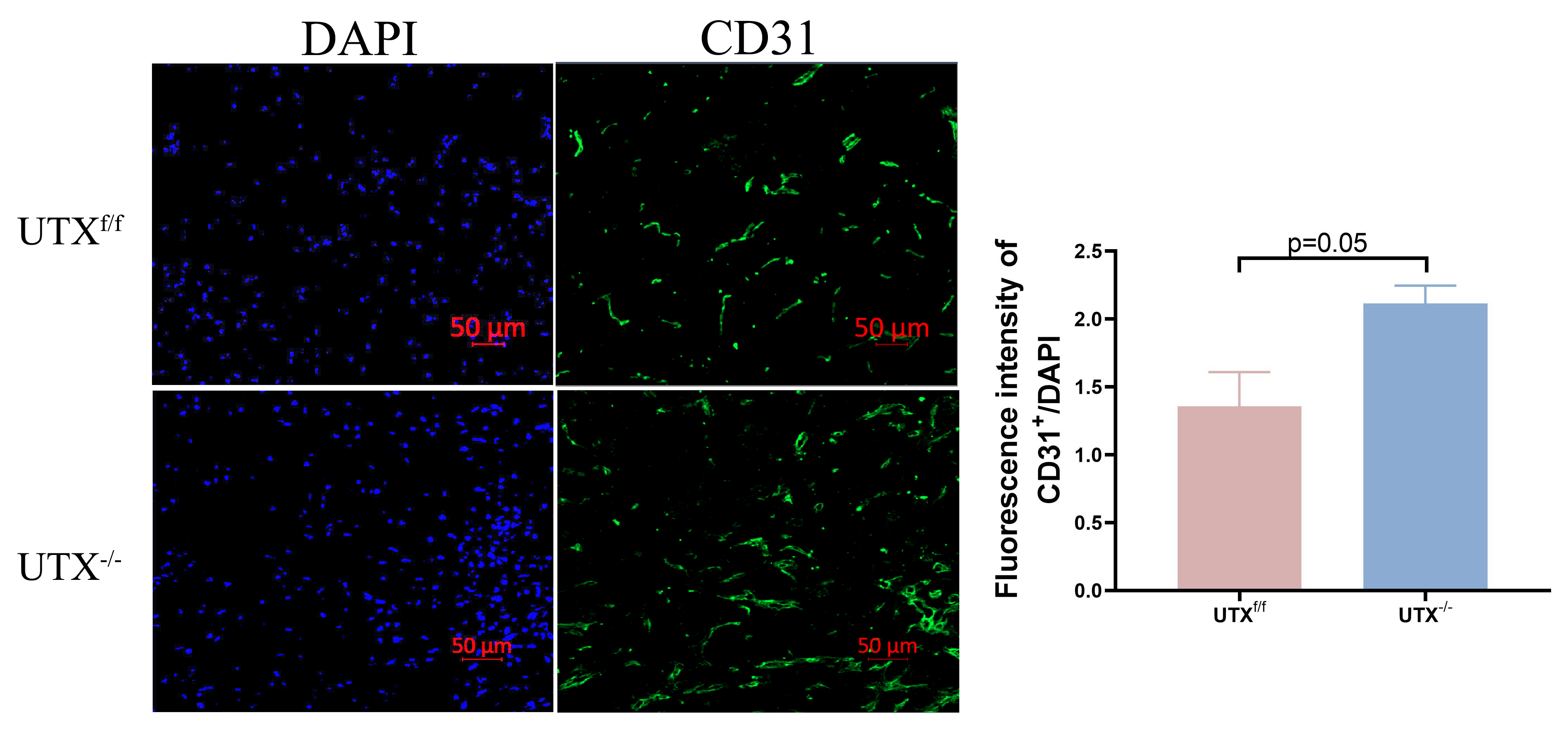

Supplement: S3 Fig — Representative images and quantitative analysis of CD31+ (green) and DAPI (blue) staining in the spinal cord of UTXf/f and UTX-/- mice. Data are presented as mean ± SEM; Scale bars: 50 μm. (TIF) [file pone.0338326.s003.tif]

Figure 4B

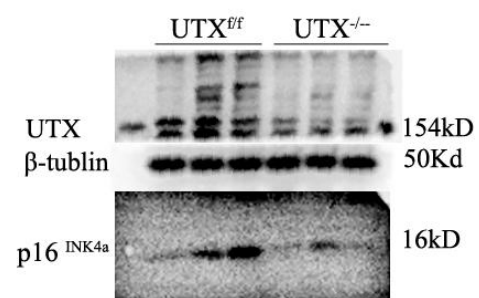

Figure 7B

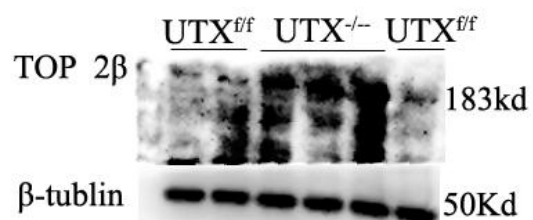

Figure 7E

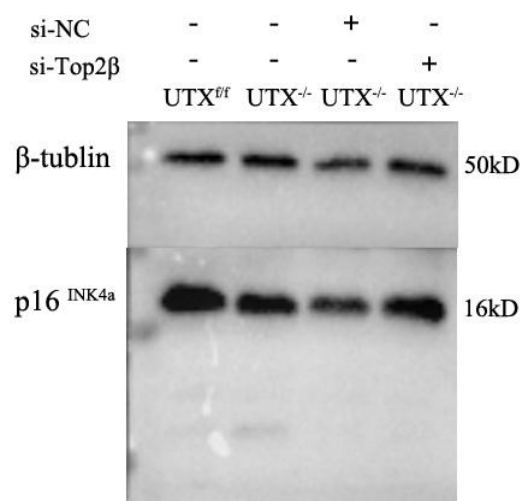

Supplement: S4 Fig — (PDF) [file pone.0338326.s004.pdf]

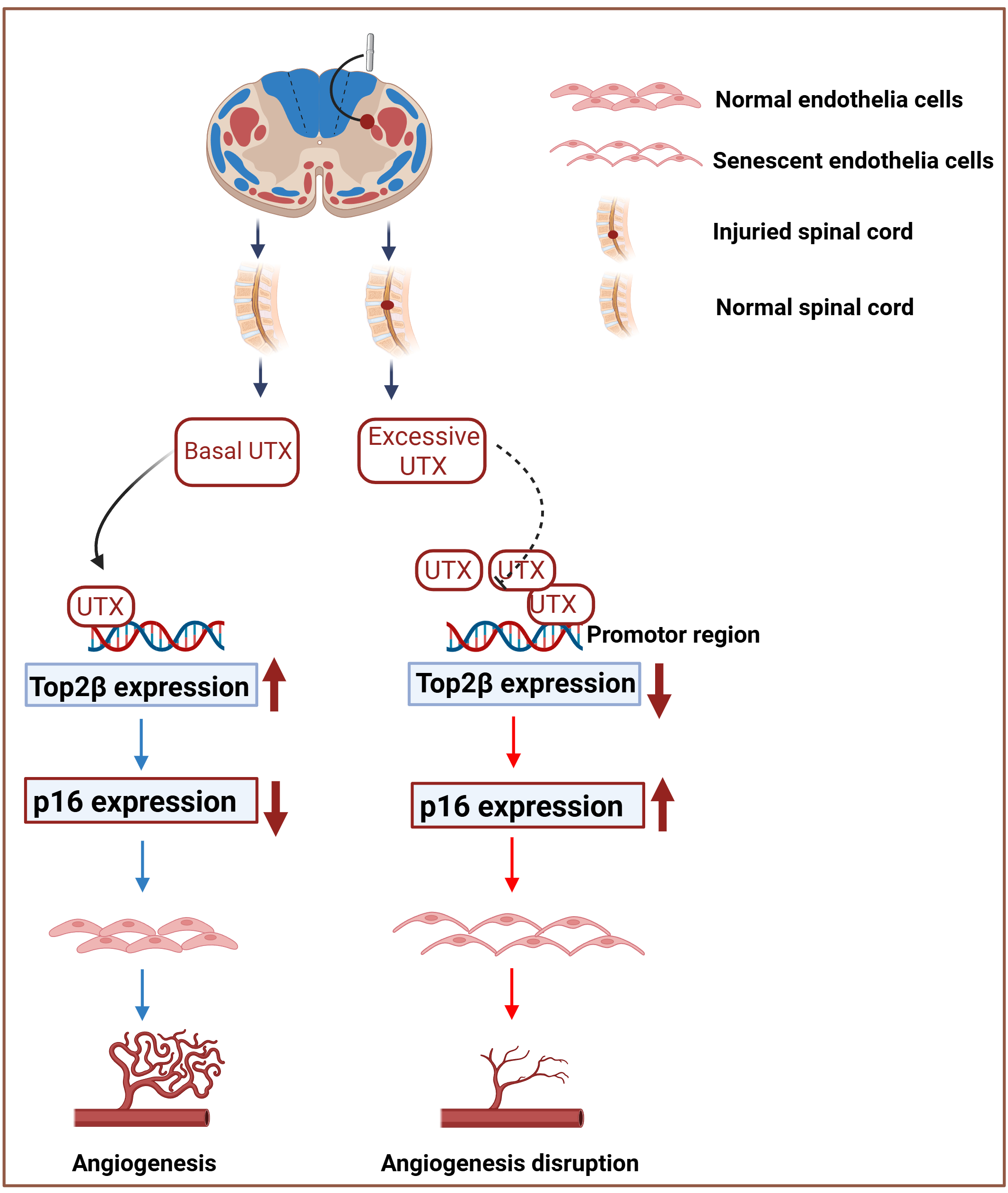

Supplement: S1 File — Schematic illustrating the proposed role of the UTX/Top2β axis in regulating spinal cord microvascular endothelial cell senescence following spinal cord injury. (TIF) [file pone.0338326.s006.tif]
